# Small Size, Big Impact: Recent Progress in Bottom‐Up Synthesized Nanographenes for Optoelectronic and Energy Applications

**DOI:** 10.1002/advs.202106055

**Published:** 2022-02-26

**Authors:** Zhaoyang Liu, Shuai Fu, Xiaomin Liu, Akimitsu Narita, Paolo Samorì, Mischa Bonn, Hai I. Wang

**Affiliations:** ^1^ University of Strasbourg CNRS ISIS UMR 7006 8 allée Gaspard Monge Strasbourg 67000 France; ^2^ Max Planck Institute for Polymer Research Ackermannweg 10 Mainz 55128 Germany; ^3^ Organic and Carbon Nanomaterials Unit Okinawa Institute of Science and Technology Graduate University 1919‐1 Tancha, Onna‐son Kunigami Okinawa 904‐0495 Japan

**Keywords:** bottom‐up synthesis, graphene nanoribbons, nanographenes, optoelectronics, van der Waals heterostructures

## Abstract

Bottom‐up synthesized graphene nanostructures, including 0D graphene quantum dots and 1D graphene nanoribbons, have recently emerged as promising candidates for efficient, green optoelectronic, and energy storage applications. The versatility in their molecular structures offers a large and novel library of nanographenes with excellent and adjustable optical, electronic, and catalytic properties. In this minireview, recent progress on the fundamental understanding of the properties of different graphene nanostructures, and their state‐of‐the‐art applications in optoelectronics and energy storage are summarized. The properties of pristine nanographenes, including high emissivity and intriguing blinking effect in graphene quantum dots, superior charge transport properties in graphene nanoribbons, and edge‐specific electrochemistry in various graphene nanostructures, are highlighted. Furthermore, it is shown that emerging nanographene‐2D material‐based van der Waals heterostructures provide an exciting opportunity for efficient green optoelectronics with tunable characteristics. Finally, challenges and opportunities of the field are highlighted by offering guidelines for future combined efforts in the synthesis, assembly, spectroscopic, and electrical studies as well as (nano)fabrication to boost the progress toward advanced device applications.

## Graphical abstract

The recent progress on the fundamental properties of different bottom‐up synthesized nanographenes, and the collective properties upon forming nanographene‐2D material‐based van der Waals heterostructures, toward their state‐of‐the‐art applications in optoelectronics and energy storage is summarized. Forthcoming challenges and opportunities of this emerging field are highlighted, and perspectives in boosting the progress toward advanced device applications are offered.

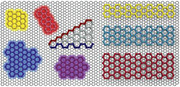

## 1 Introduction

The discovery of the outstanding properties of graphene has nurtured the fast development of atomically thin 2D materials for fundamental studies and technological advancements, which has opened a new era in nanoscience, material science, and condensed‐matter physics.^[^ ^1^ ^]^ The superior properties of graphene, including broad optical absorption and response, very high electrical and thermal conductivity, and high specific surface area, render it a fascinating material platform for cutting‐edge applications, such as ultra‐broadband photodetectors, ultrafast optical modulators, and energy conversion and storage devices.^[^ ^2^, ^3^, ^4^ ^]^ However, the inherent lack of bandgap in its electronic structure limits graphene's applications in field‐effect transistors (FETs) related logic devices, limiting its use for the booming semiconductor industry.^[^ ^5^ ^]^ Thus, opening the bandgap of graphene becomes essential for extending its applications, particularly for electronics and optoelectronics.^[^ ^6^ ^]^ In principle, bandgap opening in graphene can be achieved by introducing quantum confinement effect, e.g., by “sculpting” graphene into its nanometer‐sized structures.

**Figure 1:**
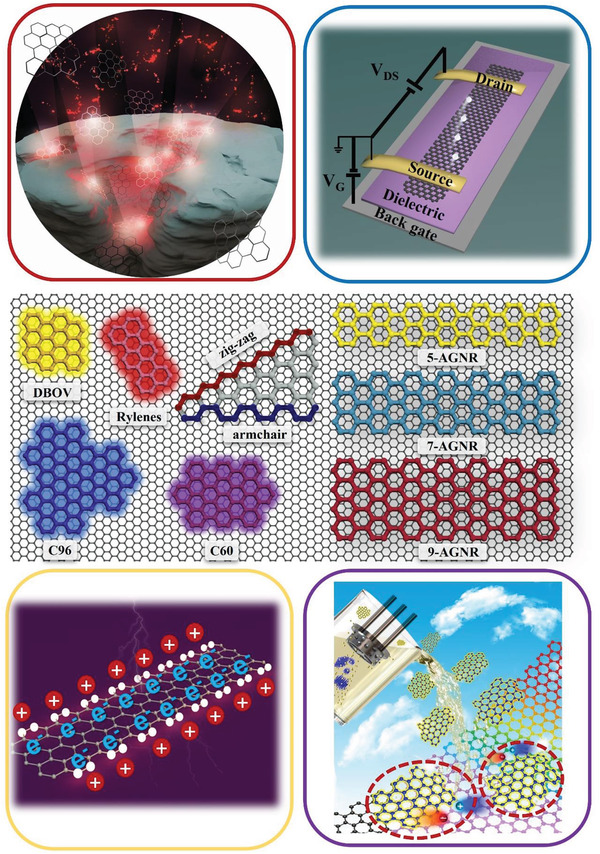
Exemplary molecular structures of bottom‐up synthesized nanographenes and their device applications: the middle panel illustrates the structures of some typical NGs. Here DBOV stands for dibenzo[*hi*,*st*]ovalene, while 5‐, 7‐, 9‐AGNR represent armchair GNRs with 5, 7, and 9 atoms in the width direction, respectively. Rylenes correspond to short 5‐AGNRs and are represented by the structure of perylene. C96 and C60 are GQDs consisting of 96 and 60 sp^2^ carbon atoms. The four panels in the conners conceptually demonstrate i) the highly emissive properties of GQDs for light emission applications (the upper left panel). Reproduced with permission.^[^ ^72^ ^]^ Copyright 2020, Wiley‐VCH.; ii) highly mobile charge carriers in GNRs for field effect transistors (the upper right panel); iii) the edge‐specific electrochemistry for ion storage or catalysis (the bottom‐left panel, with the white dots highlighting the role of specific edge effect), and iv) integrating NG‐2D material van der Waals heterostructures (highlighted by the circles) for optoelectronics applications (the bottom‐right panel).

In this minireview, we term these graphene nanostructures as “nanographenes (NGs),” and primarily focus on the optical and electronic properties of NGs with strong electronic confinement effects with at least one of their lateral dimensions being below 10 nm. The emerging NGs include “graphene quantum dots (GQDs),” which are laterally extended polycyclic aromatic hydrocarbons (PAHs), and their 1D extension, i.e., “graphene nanoribbons (GNRs),” as shown in **Figure** **1**.

NGs exhibit tunable and finite optical bandgaps (from IR to UV range), making them appealing for next‐generation, green electronics, and optoelectronics. The “green” nature and the abundance of the carbon element make NGs appealing for device applications from environmental and economic perspectives. Substantial synthesis effort has been made previously for opening and controlling the bandgap of NGs by tailoring their sizes, dimensions, and edge structures with ultimate atomic precision.^[^ ^7^ ^]^ So far, both top‐down and bottom‐up approaches have been developed. While the former method is based on breaking large carbon allotropes into nanoscale pieces, the latter focuses on assembling the precursor molecules as tiny “LEGO” pieces into the final NGs via organic synthesis, pyrolysis, or other wet chemical methods.^[^ ^8^, ^9^ ^]^ For top‐down approaches, previous attempts to fabricate NGs (e.g., GNRs) by hydrothermal cutting of graphene oxide nanosheets, photolithography patterning of graphene, and unzipping of carbon nanotubes have been reported. Yet, despite the successful demonstration of electronic bandgap opening,^[^ ^10^, ^11^, ^12^, ^13^ ^]^ the use of top‐down methods offers poor structural control in sizes below 10 nm, hence limiting the bandgap values below 400 meV.^[^ ^11^ ^]^ Furthermore, such top‐down methods give rise to poorly defined edge structures in the resultant NGs, hindering the precise control of their properties for advanced applications.

In contrast, the bottom‐up organic synthesis approach pioneered by Müllen and co‐workers^[^ ^14^, ^15^, ^16^, ^17^, ^18^, ^19^, ^20^, ^21^, ^22^, ^23^, ^24^, ^25^, ^26^, ^27^, ^28^, ^29^, ^30^, ^31^, ^32^, ^33^, ^34^, ^35^, ^36^, ^37^, ^38^, ^39^, ^40^, ^41^, ^42^, ^43^, ^44^, ^45^ ^]^ provides atomically precise control of NGs (e.g., width and edges), and consequently, fine‐tuning of their electronic properties. The organic synthesis of NGs is typically achieved by the oxidative cyclodehydrogenation of oligophenylene and polyphenylene precursors, either in solution or on a metal surface. Thus, a variety of NGs with different edge structure,^[^ ^16^, ^17^, ^18^, ^44^ ^]^ size,^[^ ^17^, ^18^ ^]^ nonplanarity,^[^ ^34^, ^36^, ^38^, ^46^, ^47^ ^]^ and/or chirality^[^ ^20^, ^32^, ^48^ ^]^ which are predefined by judiciously choosing and designing the tailor‐made precursor molecules; side chains and other functional groups can also be introduced at peripheral positions to modulate the electronic properties and solubility of NGs.^[^ ^49^ ^]^ Besides the organic synthesis approach, we note that other bottom‐up synthesis routes (e.g., for fluorescent GQDs), including pyrolysis and solvothermal methods, have been developed.^[^ ^8^, ^9^ ^]^ Despite being successfully applied, these approaches lack precise structural control at the atomic level. Here we refer readers to references,^[^ ^5^, ^6^, ^7^, ^50^, ^51^, ^52^, ^53^, ^54^, ^55^, ^56^, ^57^, ^58^, ^59^, ^60^, ^61^, ^62^, ^63^, ^64^, ^65^, ^66^, ^67^ ^]^ where recent advances in the bottom‐up synthesis have been extensively reviewed. On the other hand, a timely review of the fundamental properties of NGs and related applications is critical for their device implementation. Some recent reviews have focused mainly on the electrical properties of NGs and their integration into electronics.^[^ ^68^, ^69^, ^70^, ^71^ ^]^ Here, we summarize the most recent advances in the fundamental understanding of the optical and electronic properties of NGs (particularly those made by bottom‐up synthesis) and their applications, more generally in optoelectronics and energy storage.

We structure our review by first discussing the individual properties of NGs, with a special focus on the fascinating optical properties (e.g., highly emissive nature and intriguing blinking effect in GQDs), superior charge transport properties in GNRs, and high specific capacitance of NGs for electrochemical energy storage. Second, thanks to the strong *π*–*π* interactions within NGs and between NGs and 2D materials (e.g., graphene), supramolecular self‐assemblies at larger scales allow for control of the collective properties of the system. In particular, we will review the recent development in NG‐based van der Waals heterostructures (vdWHs), highlighting the potential of such solution‐processable and tunable hybrid strategy for versatile device applications.

## 2 Nanographenes: From Structure‐Dependent Properties to Applications

### 2.1 Highly Emissive Graphene Quantum Dots for Optoelectronics

GQDs are discrete quasi‐0D NGs with sizes typically less than 10 nm in all three spatial dimensions (see some representative structures in the middle panel of Figure 1), which chemically can be seen as extended polycyclic aromatic hydrocarbons (PAHs). In recent years, GQDs have aroused extensive interest on account of their fascinating optical properties, including high absorption coefficient (≈10^5^ cm^−1^), near‐unity photoluminescence quantum yield (PLQY),^[^ ^22^, ^30^, ^73^, ^74^, ^75^ ^]^ and tunable stimulated emission over a wide spectral range (from deep ultraviolet to near‐infrared) by engineering their shapes and edge structures.^[^ ^76^, ^77^, ^78^, ^79^ ^]^ In addition, compared to conventional heavy‐metal‐based luminescent QDs (e.g., Pb‐ and Cd‐based QDs), GQDs are non‐toxic and exhibit high stability and good solution‐processability, making them ideal candidates for developing environmentally friendly solid‐state light‐emitting sources.^[^ ^80^ ^]^

**Figure 2:**
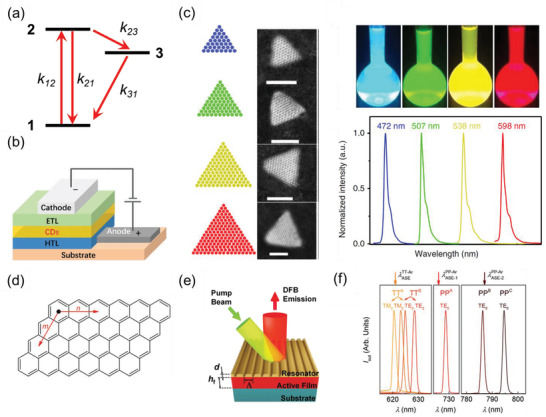
a) Schematic of a three‐level system to show the competition between radiation recombination (*k*_21_) and intersystem crossing (*k*_23_). Here state 1, 2, 3 represent the ground state, the first singlet excited state, and the lowest triplet state respectively; b) Schematic of the typical device configuration of GQD‐based electroluminescent LEDs. Reproduced under the terms of the Creative Commons CC‐BY license.^[^ ^86^ ^]^ Copyright 2021, The Authors. Published by Wiley‐VCH. c) The left panel shows schematic and aberration‐corrected HAADF‐STEM images of triangular GQDs, and the right panel presents fluorescence images of triangular GQDs under UV light and their PL spectra under 365 nm excitation. Reproduced with permission.^[^ ^9^ ^]^ Copyright 2018, Springer Nature. d) Chemical structure of [*n*,*m*]*peri*‐acenoacene, representing generic *n* by *m* four‐zig–zag edged GQD with a parallelogramic shape.^[^ ^88^ ^]^ e) Schematic of the DFB resonator. The active film (*h*, film thickness) composed of GQDs is dispersed in polystyrene and deposited on a fused silica substrate. The film is covered by a top‐layer polymeric resonator with an engraved relief grating (*Λ*, grating period; *d*, grating depth).^[^ ^88^ ^]^ f) Spectra of DFB lasers based on GQD‐doped polystyrene films: the label indicates the GQDs dispersed in the film, and letters are used to discern devices with different geometrical parameters. Each laser emission is composed of either one or two peaks (laser modes), which are associated with the waveguide mode (TE_0_ or TM_0_) and whose polarization is parallel or perpendicular to the DFB grating lines, respectively. d–f) Reproduced with permission.^[^ ^88^ ^]^ Copyright 2021, Wiley‐VCH.

Optical excitations with photon energy exceeding the energy gap of GQDs can promote electrons from the ground state to excited states, leading to absorption signatures typically distributed in the UV–vis range. These absorption signatures are mainly attributed to the *π*–π* electronic transitions of sp^2^‐hybridized carbon atoms, while n‐π* electronic transitions may also contribute, in the presence of heteroatoms with lone pairs.^[^ ^81^ ^]^ The electrons populating the excited state can undergo radiative recombination by emitting photons (with a rate of *k*_21_), or relax via intersystem crossing nonradiatively back to the ground state (following the path from the excited state **2** to the triplet state **3** and then to the ground state **1**, see **Figure** **2**a). As a figure of merit for lighting and display applications, derivatives of rylene and dibenzo[*hi*,*st*]ovalene (DBOV) (Figure 1), with a combination of zig–zag and armchair edges, have been shown to exhibit up to near‐unity PLQY.^[^ ^22^, ^30^, ^73^, ^74^, ^75^ ^]^ Their highly emissive nature can be attributed to the low intersystem crossing rate and thus a negligible transition to the triplet state. For example, Zhao et al.^[^ ^82^ ^]^ reported a more favorable rate competition for radiative recombination (*k*_R_ ≈0.28 ns^−1^) than intersystem crossing (*k*_ISC_ ≈0.025 µs^−1^) in C96 GQDs (Figure 2a), supporting the observed high emission brightness. Recent developments in the bottom‐up synthesis of atomically precise GQDs provide excellent opportunities to tune their chemical structures and optical properties,^[^ ^83^, ^84^, ^85^ ^]^ enabling many optoelectronic applications. Figure 2b presents a typical device configuration of GQD‐based electroluminescent LEDs (ELEDs), where GQD or GQD‐embedded matrix as the emission layer is sandwiched between a hole transport layer (HTL) and an electron transport layer (ETL) and electrically triggered by a bias voltage between a cathode and an anode.^[^ ^86^ ^]^ During the device operation, electrically injected charge carriers can diffuse into GQDs through TLs and recombine radiatively. Yuan et al.^[^ ^8^ ^]^ successfully prepared GQDs with different emission colors by a solvothermal method and demonstrated GQD‐based monochrome ELEDs (from blue to red) with a maximum luminance of ≈2050 cd m^−2^ and a current efficiency of 1.1 cd A^−1^, comparable to state‐of‐the‐art semiconductor GQD‐LEDs. Employing three‐fold symmetric phloroglucinol triangulogen as the precursor, Yuan et al.^[^ ^9^ ^]^ further developed a bottom‐up solvothermal synthesis approach of multicolored triangular GQDs with extremely narrow emission features (with a full width at half maximum (FWHM) of ≈30 nm) and high quantum yields up to 54–72% (Figure 2c). The narrow emission linewidth and high color‐purity of GQDs demonstrate their great potential in display‐relevant applications. Combined with previous progress in applying organic luminescent molecules for light‐emitting applications,^[^ ^87^ ^]^ these results suggest the high potential of monodisperse GQDs by organic synthesis for the ELED applications.

Along with using GQDs as the active components in ELEDs, GQDs have also been considered as optical‐gain media for lasing applications due to their low threshold, stable stimulated emission, and wavelength tunability across the visible spectrum.^[^ ^89^ ^]^ Following the observation of amplified spontaneous emission (ASE) in DBOV,^[^ ^22^ ^]^ effects of edges and substituents have been systematically investigated,^[^ ^88^, ^90^, ^91^ ^]^ with a particular focus on four‐zig–zag (FZ) edged GQDs, or [*n*, *m*]*peri*‐acenoacene, due to their low threshold and high photostability. Figure 2d depicts the chemical structure of FZ‐edged GQDs, with a number *m* of [*n*]acenes in a parallelogramic lattice. By varying *m* or *n* while fixing the other, it has been demonstrated that 1D conjugation extension (e.g., by increasing *n*) is more efficient in tuning the PL emission wavelength to the infrared range than 2D conjugation extension.^[^ ^22^, ^88^ ^]^ Figure 2e illustrates the typical device architecture of GQD‐based distributed feedback (DFB) lasers, where a polymeric resonator with 1D relief grating is deposited on top of the GQD‐doped polystyrene films. As shown in Figure 2f, the fabricated DFB laser shows emissions at either one or two different wavelengths. Notably,^[^ ^2^, ^5^ ^]^ *peri*‐acenoacene exhibits dual‐ASE at 726 and 787 nm, respectively, promising for low‐cost near‐infrared lasing applications.^[^ ^88^ ^]^

Besides conventional optoelectronic applications, GQDs can also be considered single‐photon emitters thanks to their excellent photochemical stability and low cost, holding great promise for next‐generation quantum technologies such as quantum cryptography and communication.^[^ ^30^, ^82^, ^92^ ^]^ To this end, further progress is required to explore GQDs with suppressed blinking and high brightness to realize the controllable quantum correlations. In addition, the designable molecular orbitals in GQDs may serve as tailor‐made energy windows to extract hot carriers in sensitizers and minimize the energy loss upon light harvesting, as a proof‐of‐concept of hot carrier solar cells.^[^ ^93^, ^94^, ^95^ ^]^

#### 2.1.1 Fluorescence Blinking in Nanographenes for Super‐Resolution Imaging Applications

The fascinating combination of strong and tunable fluorescence^[^ ^72^, ^91^, ^96^, ^97^ ^]^ with high chemical and environmental stabilities^[^ ^30^ ^]^ makes GQDs also suitable for optical imaging applications.^[^ ^76^ ^]^ Moreover, compared to broad PL spectra due to a wide size distribution of NGs produced by top‐down synthesis,^[^ ^77^, ^98^, ^99^ ^]^ atomically‐precise structures of bottom‐up synthesized GQDs with monodispersed sizes enable extremely narrow PL spectra.^[^ ^22^, ^29^, ^72^, ^74^, ^75^ ^]^ For instance, DBOV and its derivatives exhibit PL spectra with a FWHM of only 15–20 nm, rendering them ideal for multi‐color imaging applications. On top of that, similar to the widely used organic dyes, grafting various functional groups (e.g., water‐soluble or other specific ligands) to GQDs enables introduction of new functionalities and biocompatible conditions for bioimaging applications.^[^ ^100^, ^101^, ^102^, ^103^ ^]^

Previously, GQDs have been considered to possess stable fluorescence properties,^[^ ^82^, ^99^, ^104^ ^]^ in contrast to the fluorescence bleaching effect that is typically observed in organic dyes (e.g., Alexa 647).^[^ ^72^ ^]^ The stable fluorescence can be readily applied in conventional wide‐field and confocal microscopy imaging applications.^[^ ^105^ ^]^ However, the spatial resolution of such conventional microscopy is diffraction‐limited to, at best, 200 nm laterally and 600 nm axially in the visible range. The recent development of super‐resolution microscopy (SRM) techniques has enabled visualizing structures with nanometric resolution,^[^ ^106^ ^]^ leading to important breakthroughs in biology and materials science. In general, SRM can be realized mainly in two ways: stimulated emission depletion (STED) microscopy^[^ ^107^ ^]^ and single‐molecule localization microscopy (SMLM).^[^ ^108^ ^]^ In particular, SMLM is an approach that achieves super‐resolution by isolating emitters from diffraction‐limited image sequences and fitting their images with the point spread function,^[^ ^109^ ^]^ and therefore requires photoswitchable or “blinking” fluorophores.

**Figure 3:**
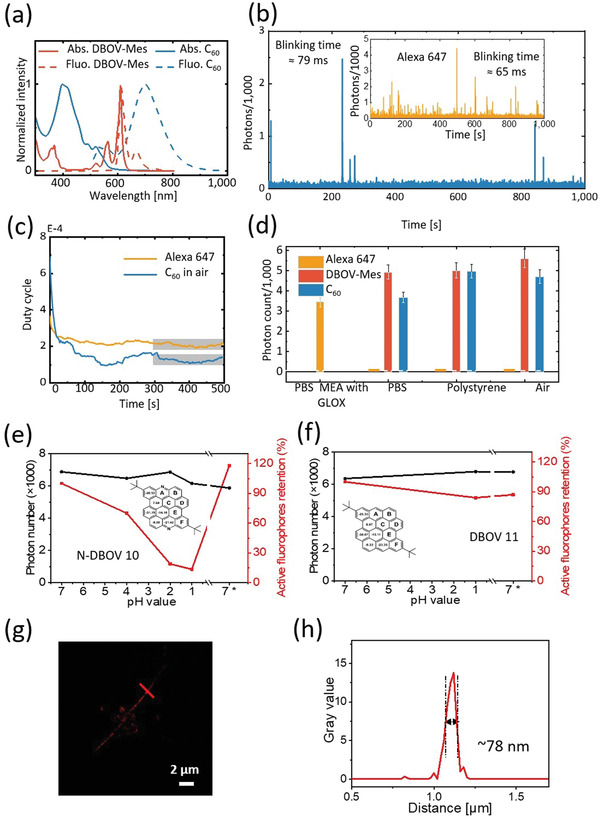
Fluorescence blinking properties of GQDs. a) PL spectra of two selected GQDs, DBOV, and HBC C60. b) Representative single‐molecule fluorescence time trace of C60 measured in air and organic dye Alexa 647 (inset) in a standard blinking buffer. c) On–off duty cycle. d) Measured photon numbers in different environments. Environmental pH‐dependent blinking properties of e) DBOV and f) N‐DBOV. g) Super‐resolution SMLM image of nanometer‐sized crevices in a glass substrate. h) Intensity profile along the red line shown in (g). a–d) Reproduced with permission.^[^ ^72^ ^]^ Copyright 2020, Wiley‐VCH. e–h) Reproduced under the terms of the Creative Common CC BY license.^[^ ^75^ ^]^ Copyright 2021, The Author, published by American Chemical Society.

In 2020, Liu et al.^[^ ^72^ ^]^ reported the first observation of the blinking effect in GQDs at the single‐molecule level (based on DBOV) under different environments, enabling their applications for SMLM. For SMLM imaging, two fluorescence blinking parameters, i.e., photon numbers (detected photons per switching event) and on–off duty cycle (the fraction of time for a molecule residing in its fluorescent “on” state), are critical for determining the imaging qualities.^[^ ^110^ ^]^ High photon numbers provide high imaging resolution, while a low on–off duty cycle could improve both the imaging accuracy and labeling density by decreasing the probability of two fluorophores fluorescing simultaneously within the diffraction‐limited imaging area. Two selected GQDs, i.e., DBOV bearing two mesityl groups (termed as DBOV‐Mes) and C60 (PAHs consisting of 60 sp^2^ carbon atoms; see the aromatic core structures in the middle panel of Figure 1), were found to have excellent blinking properties (see **Figure** **3**a–d): high photon numbers up to several thousand and low on–off duty cycle of ≈10^−4^ have been achieved in different environments (including in air, embedded in a polystyrene (PS) film, and in water). Such blinking features enable imaging of samples labeled with GQDs with nanometer‐range spatial resolution in different environments. One imaging example, nanometer‐sized crevices in a glass substrate imaged in air, is demonstrated in Figure 3g,h (with a spatial resolution below 80 nm). As a comparison, for organic dye Alexa 647, the current gold standard fluorophore for SMLM, blinking properties could only be realized in optimized blinking buffers. Most recently, a unique, stimuli‐responsive GQD, i.e., N‐DBOV, has been developed by incorporating nitrogen atoms into the zig–zag edges of DBOV.^[^ ^75^ ^]^ The synthesis of N‐DBOV was carried out in a similar manner to the improved synthetic protocol for the pristine DBOV, through the photochemical cyclodehydroiodination,^[^ ^30^ ^]^ and the nitrogen‐incorporated zig–zag edges were finally formed by the oxidative cyclization of preinstalled amino groups. The fluorescence properties of N‐DBOV are sensitive to protons (pH) as well as specific metal ions, such as Cu^2+^ and Fe^2+^ ions. Moreover, N‐DBOV also demonstrates pH‐dependent blinking properties, as shown in Figure 3e. As a comparison, its parent NG, i.e., DBOV, shows pH‐independent blinking features, as shown in Figure 3f. The combination of SMLM with the pH‐dependent blinking properties of N‐DBOV can therefore provide a sensitive probe for determining pH differences at the nanoscale.

As a brief summary of this section, GQDs exhibit outstanding photophysical properties for optical imaging applications. Significantly, the environment‐independent blinking properties pave the way to develop GQDs as a new series of fluorophores for optical super‐resolution imaging. With the incorporation of nitrogen atoms, the synthesized GQDs can be used as nanoscale sensors for pH or specific metal ion detection. These examples illustrate the golden opportunity for organic chemists to design and synthesize new GQDs structures to push this young and dynamic field forward, e.g., for achieving bioimaging. The work published so far has focused on hydrophobic GQDs, which are most suitable for material imaging. As for bioimaging applications, hydrophilic GQDs with various functional groups for realizing specific sample binding functionality are required. Such synthesis could be further inspired by Müllen's work on functionalizing rylene‐type fluorophores.^[^ ^101^, ^111^, ^112^, ^113^, ^114^, ^115^ ^]^ Meanwhile, elucidating the blinking mechanism is crucial for both fundamental and application‐oriented research areas. As for organic dyes, the blinking mechanism remains elusive and is challenging to study due to the complex buffer‐dependence and continuous chemical reactions of the buffer solutions.^[^ ^116^, ^117^ ^]^ On the other hand, the environment‐independent blinking properties of GQDs offer a great opportunity for achieving a fundamental understanding of the blinking mechanism in GQDs.

### 2.2 Graphene Nanoribbons: Excellent Charge Transport Properties and Strong Exciton Effects

While GQDs with tunable bandgaps are useful for emission and imaging applications thanks to their fascinating optical properties, their applications in electronics are restricted because of their strong spatial confinement in all three directions. Graphene nanoribbons (GNRs), the 1D extension of GQDs, possess not only finite and tunable bandgaps (by controlling the width of the ribbon), but also provide an elongated electrical transport channel for charge carriers with high electrical mobility inherited from graphene. Besides their width dimension,^[^ ^17^, ^118^ ^]^ the edge structures^[^ ^27^, ^119^, ^120^, ^121^, ^122^, ^123^ ^]^ substantially affect the electronic and thus electrical properties of GNRs. The most popular edge structures include zig–zag and armchair (see Figure 1). Armchair GNRs have been widely investigated due to their high stability and large tuning of bandgaps from IR to UV–vis. Zig–zag GNRs with their fascinating localized edge states have been considered as a promising platform for spintronics, but their synthesis and practical applications are currently impeded by their limited chemical stability.^[^ ^60^, ^124^ ^]^ There are comprehensive summaries of the advances and challenges in synthesis,^[^ ^5^, ^6^, ^7^, ^50^, ^51^, ^52^, ^53^, ^54^, ^55^, ^56^, ^58^, ^59^, ^65^, ^66^, ^67^ ^]^ structure–electronic property correlation,^[^ ^6^, ^57^ ^]^ device integration,^[^ ^71^, ^125^ ^]^ and applications in, e.g., quantum electronics.^[^ ^126^ ^]^ This section aims to overview recent advances in electrical and optical studies of GNRs, and highlights their potential impacts on optoelectronics which have rarely been covered in the previous reviews.^[^ ^4^ ^]^

**Figure 4:**
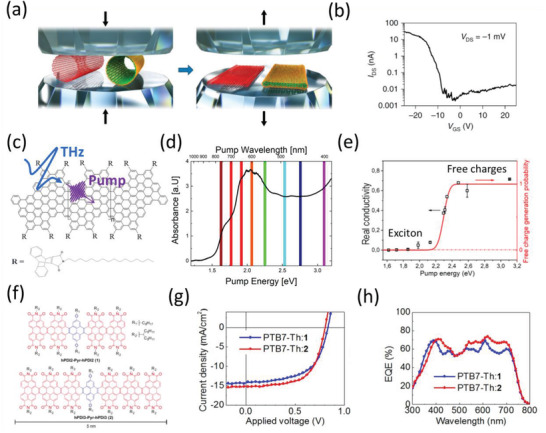
Electrical and optical properties of GNRs, and their applications in organic optoelectronics. a) Schematic presentation of fabrication of GNRs by squashing an SWCNT and DWCNT (left) into edge‐closed double‐layer and four‐layer GNRs (right) via a high‐pressure (*P*) and thermal treatment. The black arrows show the movement directions of the diamond anvils. b) characteristics of the GNR‐FET at room temperature. c) Sketch of the GNR–AHM and optical‐pump THz‐probe spectroscopy to study the exciton dynamics in GNRs. d) UV/Vis absorption spectrum of GNR–AHM in toluene. e) Pump photon energy‐dependent peak real conductivity unveils a transition from “exciton gas” to transient free carrier states. f) Two ribbons designed and synthesized: 1) hPDI2‐Pyr‐hPDI2 and 2) hPDI3‐Pyr‐hPDI3. g) J−V curves for 1) hPDI2‐Pyr‐hPDI2 and 2) hPDI3‐Pyr‐hPDI3. h) EQE spectra for 1) hPDI2‐Pyr‐hPDI2 and 2) hPDI3‐Pyr‐hPDI3. a,b) Reproduced with permission.^[^ ^128^ ^]^ Copyright 2021, Springer Nature. c–e) Reproduced under the terms of the Creative Common CC BY license.^[^ ^155^ ^]^ Copyright 2020, Published by American Chemical Society. f–h) Reproduced with permission.^[^ ^160^ ^]^ Copyright 2017, Wiley‐VCH.

Over the last decade, Dai and his colleagues pioneered the top‐down synthesis and electrical characterizations of single GNRs with diameters of sub‐10 nanometer by exfoliating and etching graphite,^[^ ^11^ ^]^ unzipping multiwalled carbon nanotubes via plasma etching^[^ ^127^ ^]^ or sonication.^[^ ^13^ ^]^ Very recently, by directly quashing carbon nanotubes via high‐pressure treatment (see **Figure** **4**a), they fabricated the closed edge GNRs with a width down to ≈3 nm. These GNRs demonstrated an extremely high charge carrier mobility *μ* approaching 2500 cm^2^ V^−1^ s^−1^ and an on–off ratio over 10^4^ (see Figure 4b), suggesting the relevance of GNRs for high‐mobility electronics.^[^ ^128^ ^]^ The bottom‐up solution synthesis of GNRs was pioneered by the Müllen group from the beginning of this century,^[^ ^129^, ^130^ ^]^ through the extension of their NG synthesis to polymers, applying the highly efficient oxidative cyclodehydrogenation conditions to tailor‐made polyphenylene precursors.^[^ ^7^, ^26^, ^61^, ^64^, ^70^ ^]^ In 2010, Müllen and Fasel demonstrated an on‐surface synthesis of GNRs by performing the polymerization and cyclodehydrogenation reactions on a gold surface under ultrahigh vacuum conditions, enabling the in‐situ visualization of the atomically precise chemical structures of GNRs using high‐resolution scanning‐probe microscopes.^[^ ^5^, ^6^, ^7^, ^63^, ^66^, ^131^, ^132^ ^]^ The key has been the design and synthesis of dihalogenated monomers that determine the resulting GNR structures, and a variety of GNRs have been synthesized over the last decade, revealing a wide range of structure‐dependent exotic electronic properties, including topological and spin‐polarized electronic states.^[^ ^27^, ^124^, ^131^, ^133^, ^134^, ^135^, ^136^ ^]^ Electrical characterizations of bottom‐up synthesized single GNRs (via solution or on‐surface synthesis) remain relatively rare and challenging,^[^ ^68^, ^71^, ^137^, ^138^, ^139^, ^140^, ^141^, ^142^ ^]^ partially due to the ribbons’ relatively short length by bottom‐up approaches and large contact resistance.^[^ ^143^, ^144^, ^145^, ^146^ ^]^ In this regard, contact‐free photoconductivity measurements of GNRs, e.g., by terahertz (THz) spectroscopy, provide a complementary and powerful tool to characterize the charge transport properties of GNRs (in particular the short ones with sub‐100 nm length).^[^ ^147^ ^]^ In typical THz measurements, transiently free charge carriers are optically injected into GNRs by a short laser pulse (with a duration of ≈100 fs), and their conductivity is probed by an oscillating THz field with a duration of ≈1 ps (see Figure 4c). Due to the ac nature of the probe, THz measurements provide direct access to the intrinsic, local intra‐GNR charge transport properties (e.g., over a length scale of 10 s of nm). In line with the electrical measurements for the top‐down synthesized GNRs, THz studies unveil the high charge carrier mobility of varied solution‐synthesized GNRs, in the range 100–1000 cm^2^ V^−1^ s^−1^.^[^ ^18^, ^26^, ^46^ ^]^ Intriguingly, over an extensive range of GNRs studied, a “universal” charge scattering time (*τ*) of ≈30 fs is observed regardless of their lengths and synthesis methods, suggesting a likely role of structural distortion on a length scale of ≈20 nm in limiting the mobility of charge carriers in the GNRs dispersed in solution.^[^ ^18^, ^46^, ^148^, ^149^ ^]^ Subsequently, controlling the charge carrier effective mass (m^*^) via tuning the width,^[^ ^17^, ^118^ ^]^ and flatness^[^ ^36^ ^]^ and edge structures^[^ ^46^ ^]^ has proven a fruitful direction to tune the carrier mobility (*μ* $=\frac{e\tau }{m^{*}}\;$) in solution‐synthesized GNRs.

Besides providing direct access to local charge transport properties, the second fascinating feature of GNRs revealed by spectroscopic measurements is their exciton dominant optical properties, prevailing even at room temperature. Following optical excitations, photogenerated electrons and holes in GNRs are subjected to strong Coulomb interactions, resulting in tightly bounded electron−hole pairs, i.e., excitons, which have been theoretically predicted by several groups.^[^ ^150^, ^151^, ^152^, ^153^ ^]^ Employing reflectance difference spectroscopy, Ruffieux and co‐workers^[^ ^154^ ^]^ identified and confirmed that the excitonic nature of the optical transitions governs the optical transients with large and anisotropic transition dipoles in narrow armchair edged GNRs with seven atoms in width (7‐AGNRs). By tuning the excitation photon energies, Wang and co‐workers^[^ ^155^ ^]^ have recently shown that they can selectively populate and distinguish the “insulating” exciton states from the conductive free carriers. This allowed quantifying the exciton binding energy, i.e., the energy threshold required to ionize excitons into free electrons and holes, of 7‐AGNRs to be 700 meV (see Figure 4d,e).^[^ ^155^ ^]^ Other exciton species (including charge excitons^[^ ^153^ ^]^ and biexcitons^[^ ^79^ ^]^) have also been proposed to play a critical role in the optical properties of GNRs, rendering the materials as a novel platform for studying exciton physics and varied optoelectronic applications (e.g., “excitonic” solar cells^[^ ^156^ ^]^).

Along with the strong exciton effect, the fluorescence properties of GNRs have also received considerable attention in the past decade. For example, using combined atomic force microscopy and confocal fluorescence microscopy, Zhao et al.^[^ ^157^, ^158^ ^]^ characterized the fluorescence properties of GNRs in the forms of suspension and film, demonstrating the ability of GNRs to emit light in the solid‐state form. Recently, Yao et al.^[^ ^46^ ^]^ reported that fjord‐edged GNRs dispersed in tetrahydrofuran showed a red‐shifted emission with respect to the constituted model compounds due to the extension of the *π*‐conjugation. The relatively broad emission covering 550–850 nm could be potentially appealing for optoelectronic applications (e.g., white light emitting diode). To further access the fluorescence properties of individual GNRs, Chong et al.^[^ ^140^ ^]^ suspended an individual 7‐AGNR between the tip of a scanning tunneling microscope and a Au(111) surface. The electroluminescence spectra of such GNR junctions exhibited a narrow emission, whose energy can be readily controlled by varying the applied bias voltage. In combination with ab initio calculations, the narrow emission was assigned to intra‐GNR excitonic transitions involving the localized states at the GNR termini (i.e., so called Tamm states) and the delocalized electronic states along the GNR. Furthermore, Senkovskiy et al.^[^ ^159^ ^]^ found that blue laser irradiation in ambient conditions or hydrogenation in ultrahigh vacuum can greatly enhance the PL intensity of 7‐GNRs by generating sp^3^ defects, demonstrating the potential impact of defect engineering for tuning and enhancing the PL properties of GNRs. Along this line, further investigating the roles of edge structure and length in the fluorescence properties of individual GNRs, and the effects of defect nature and densities on the fluorescence properties of GNRs is critical to achieve fine tuning of their emission properties toward advanced light emitting applications.

#### 2.2.1 Graphene Nanoribbons as Highly Conductive Organic Conductors for Efficient Optoelectronics

By uniquely combining high intrinsic mobility and strong exciton effect (featuring strong light absorption), GNRs can be seen as highly conductive organic semiconductors,^[^ ^26^ ^]^ which hold great potential for organic optoelectronics, e.g., organic photovoltaics (OPV). In this direction, Beljonne and co‐workers^[^ ^161^ ^]^ modeled the electronic and optical properties of various GNRs using (time‐dependent) DFT calculations, and established the GNRs‐fullerene (donor–acceptor) heterojunctions for efficient OPVs. In a follow‐up theoretical work by Meunier and co‐workers,^[^ ^162^ ^]^ GNR‐based heterojunctions with tunable type‐II staggered band alignments (by doping) have been predicted to deliver a high power conversion efficiency up to 22.0%. In 2017, Nuckolls and co‐workers^[^ ^160^ ^]^ implemented GNRs as exceptional electron acceptors for OPV, and demonstrated efficiencies of ≈8% by employing PTB7‐Th as electron donors (see Figure 4f–h). Besides utilizing GNRs as the active layer materials, they can also be employed as interfacial charge‐transporting layers, owing to their high charge carrier mobility and solution‐processability. For instance, Nuckolls and Echegoyen^[^ ^163^ ^]^ demonstrated that GNRs can act as efficient electron‐transporting layers in perovskite solar cells to achieve ≈17% efficiency (in comparison to the by‐then record efficiency of ≈20%), outperforming commonly used PC_61_BM. Finally, by exploiting the high dispersibility of solution‐processed GNRs, Samorì and co‐workers^[^ ^164^ ^]^ demonstrated that the addition of 18 atoms wide armchair GNRs into a P3HT matrix enables significant improvement in the charge transport properties of the P3HT FET devices, without altering the I_on_/I_off_ ratio. This can be attributed to GNRs providing percolation paths for the charge carrier transport by connecting the P3HT domains. The subsequent photoresponse of such GNR‐P3HT blend film indicates the great potential of GNRs for organic (opto‐)electronics and organic solar cells.

While the studies showcased above illustrate the great potential of bottom‐up GNRs for organic optoelectronics, their full potential has not been exploited so far. Despite great progress in bottom‐up synthesis in the past ten years, many open questions remain on the fundamental investigation of the unique and fascinating physical properties, e.g., localized, spin‐polarized edge states,^[^ ^28^, ^33^ ^]^ and topological edge states.^[^ ^27^, ^122^, ^123^ ^]^ Application‐oriented studies for GNRs, which are attractive and interesting by themselves, lag far behind. Up to now, one of the potential bottlenecks for applications seems to lie in the synthesis, where a large‐scale fabrication on the order of gram quantities is challenging;^[^ ^165^ ^]^ the length of GNRs, despite some exemplary success,^[^ ^26^ ^]^ is typically limited in the range of sub‐100 nm, partially due to the low solubility of longer GNRs. On top of that, the limited processability of GNRs is also a key issue, prohibiting the achievement of highly efficient devices that fully utilize the fascinating intrinsic properties of GNRs. In this regard, besides designing various new GNR structures, further research efforts aimed at large‐scale synthesis and improving processing methods (e.g., to avoid aggregation) are key toward implementing GNRs for optoelectronic devices.

### 2.3 The Unique Edge‐Specific Electrochemistry for Efficient Energy Storage

**Figure 5:**
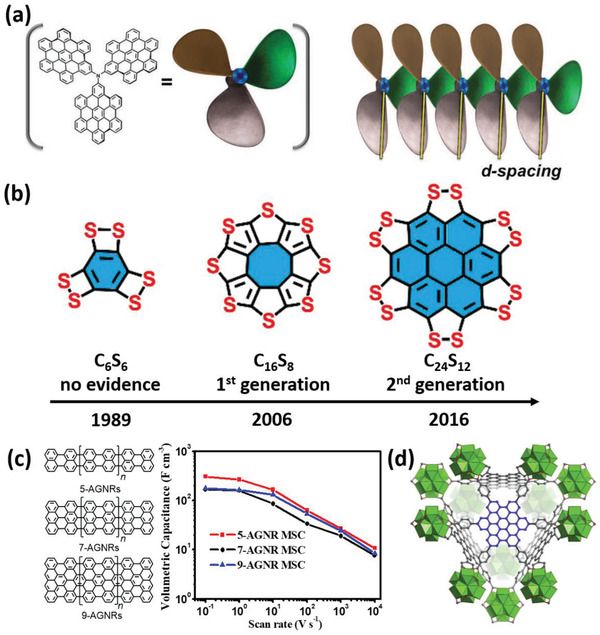
NGs for efficient energy storage. a) Schematic representation of triarylamine‐based NGs self‐assembles into highly ordered structures by stacking one flake on top of another, d‐spacing between NG flakes is represented as the distance between the two lines. Reproduced with permission.^[^ ^173^ ^]^ Copyright 2016, Wiley‐VCH. (b) “Sulflower” molecules: (left) persulfurated benzene (C_6_S_6_); (middle) fully sulfur‐substituted circulene (C_16_S_8_); (right) persulfurated coronene (C_24_S_12_). Reproduced with permission.^[^ ^19^ ^]^ Copyright 2017, American Chemical Society. (c) Molecular structures and electrochemical performance of 5‐AGNR, 7‐AGNR, and 9‐AGNR. Reproduced with permission.^[^ ^118^ ^]^ Copyright 2020, American Chemical Society. (d) The 3D structure of MOFs implanted with GQDs. Reproduced with permission.^[^ ^178^ ^]^ Copyright 2020, Elsevier.

Thanks to its broad electrochemical potential window, high intrinsic areal capacitance, and superior electrical and ionic conductivity, graphene has been widely utilized for energy conversion and storage, such as lithium‐ion batteries (LIBs) and micro‐supercapacitors (MSCs).^[^ ^166^, ^167^, ^168^, ^169^ ^]^ Li ions can adsorb on both sides of graphene, leading to significantly improved specific charge capacity as anode materials for energy storage.^[^ ^170^ ^]^ Intriguingly, the edge of single‐layer graphene, despite accounting for only a small fraction of the total “surface,” has been demonstrated to deliver 10 000 times higher specific capacitance (10^5^ µF cm^−2^ of edge electrode over 4 µF cm^−2^ of basal‐plane electrode) and significantly faster electron transfer rates than those of graphene basal planes.^[^ ^171^ ^]^ Furthermore, the open edges of graphene have been reported to enhance the ion penetration into the whole microelectrodes, leading to outstanding specific capacitance.^[^ ^172^ ^]^ Therefore, NGs combining many open edges and the excellent conductivity for electrical charges (i.e., electrons and holes) and ions represent a new class of candidates for electrochemical energy storage. Moreover, the possibility of heteroatom doping at the edges of NGs by utilizing the toolbox of organic synthesis offers various opportunities to tune and study the contribution of edge‐specific electrochemistry for high‐performance energy conversion and storage systems, i.e., batteries, supercapacitors and electrocatalysts. Along these lines, a few recent reports have shown the power of the “edge‐specific electrochemistry” for efficient energy storage. For instance, Wu and co‐workers^[^ ^173^ ^]^ have demonstrated bottom‐up synthesized 3D triarylamine‐based NGs with various functional groups on the edge of the NG flakes (**Figure** **5**a), which allow finely tunable electron density on each NG flake and distance between the adjacent flakes. This enables optimized geometric and electronic structures for efficient Li incorporation/extraction and diffusion. A high capacity of 950 mAh g^−1^ is achieved. This value is three times higher than that of conventional graphite anodes (372 mAh g^−1^), where the majority of ions are expected to be stored in the basal plane. In addition, the *π*–*π* interaction between different NG flakes has been claimed to form a robust self‐assembled nanostructure, enhancing the long‐term charging/discharging stability. Feng and co‐workers^[^ ^19^ ^]^ reported the synthesis of a persulfurated NG as the next‐generation “sulflower,” which consisted of all‐sulfur periphery and a coronene core (Figure 5b). The sulfur‐rich nature of the achieved “sulflower” translates into a high capacity of 520 mAh g^−1^, when employed as the cathode material for lithium‐sulfur batteries. Kang and co‐workers^[^ ^174^ ^]^ have reported organic anode materials based on fluorinated‐contorted NG, which can be used for both Li/Na ion batteries with a maximum specific capacity of 160 mAh g^−1^. Based on DFT calculations, they attributed the enhanced battery performance to the energetically favorable adsorption of Li/Na ions in the empty space between fluorine atoms and curved aromatic rings. The same group later reported the contorted nanographene: fullerene organic cocrystal as an anode material for conducting agent‐free LIBs, with improved specific capacity reaching 330 mAh g^−1^ (much higher than that of the pristine contorted nanographene, 80 mAh g^−1^).^[^ ^175^ ^]^ The C‐S bonds at the edges of nanocarbon materials are important active sites for energy storage, which have a great impact on the electrical double layer and introduce pseudocapacitance to the materials. Wu et al.^[^ ^176^ ^]^ demonstrated the fabrication of continuous ultrathin sulfur‐doped graphene (SG) films, based on bottom‐up synthesized peripherical trisulfur‐annulated hexa‐*peri*‐hexabenzocoronene (SHBC). The MSCs fabricated on sulfur‐doped graphene films deliver a remarkably high volumetric capacitance of 582 F cm^−3^ (much higher than that of reduced graphene, 245 F cm^−3^), and ultrahigh power density of 1191 W cm^−3^. Finally, besides the contributions of edges to the fast ion transport and storage, the overall electrical conductivity has been shown to play a critical role in affecting the overall electrochemical charging process. To better utilize the open edge structures of graphene for energy storage with atomic precision, Müllen and co‐workers^[^ ^118^ ^]^ reported micro‐supercapacitors based on bottom‐up‐synthesized armchair GNR (AGNR) films (Figure 5c). The micro‐device delivered an excellent volumetric capacitance and ultra‐high power density, which can be correlated with the charge carrier mobility within the employed GNRs, as elucidated by THz spectroscopy. Along these lines, Samorì et al. very recently reported a facile and scalable approach to exfoliate the GNRs to yield individual nanoribbons by untying the bundles of solution synthesized GNRs using a high‐shear mixing approach.^[^ ^177^ ^]^ Partially thanks to the preserved excellent electrical properties of GNRs, micro‐supercapacitor devices built on these solution‐processed GNRs deliver a high volumetric capacitance of 355 F cm^−3^ (in comparison to that of electrochemically exfoliated graphene with volumetric capacitance below 100 F cm^−3^) and a high power density of 550 W cm^−3^, thus demonstrating the great promise of GNR electrodes for energy storage again. In addition, Yu and coworkers^[^ ^178^ ^]^ reported microporous and stable zirconium‐based metal‐organic frameworks (MOFs) which implanted *π*‐conjugated GQD moieties via post‐modification of the organic ligand. Introducing GQD within the MOFs significantly improves the electrical conductivity of the composites, leading to an outstanding specific capacitance of 694 F g^−1^ (as compared to the sample without NG moieties, 269 F g^−1^), which is highly promising for supercapacitors (Figure 5d).

Besides energy storage applications, the full accessibility and incorporation of abundant heteroatoms (e.g., N, B, O, S, F, P) into NG edges offer unique opportunities to modulate the edge activity toward chemical energy production reactions, e.g., via electrocatalysts. For instance, Li and co‐workers^[^ ^179^ ^]^ synthesized the GQD‐rhenium complex as an efficient electrocatalyst and photocatalyst to selectively reduce CO_2_ into CO at an unusually low negative potential (−0.48 V vs NHE). Furthermore, bottom‐up synthesized GNRs with nitrogen atom doping at the edges were employed for constructing Fe‐Nx active sites in Fe/N/C catalysts, which delivered excellent ORR activities.^[^ ^180^ ^]^ Noteworthy, the catalytic results indicate the overall ORR activity is proportional to the number of Fe‐Nx active sites formed at the edges of GNRs, and the efficiency of the formation of Fe‐Nx active sites is mainly determined by the position of nitrogen atoms in the framework of N‐GNR precursor.

Overall, the examples discussed above demonstrate the power of “edge engineering” toward efficient electrochemical storage and catalytic reactions for chemical energy production. To date, structurally defined NGs have still not been fully explored for advanced energy conversion and storage systems. Future effort toward optimizing NG synthesis with ad hoc design concepts, e.g., large‐scale solution synthesis and processing of ultralong, highly conductive GNRs with fewer defects, higher solubility, and stable zig–zag edge structures, may open up new possibilities for this emerging class of materials.^[^ ^118^, ^181^ ^]^ Moreover, as emerging advanced rechargeable batteries, sodium‐ion batteries (SIB) have been extensively studied in recent years due to their low cost and the natural abundance of sodium, together with the low reduction potential (−2.71 V vs the standard hydrogen electrode (SHE)) that imparts safety to the SIBs.^[^ ^182^ ^]^ Because the radius of sodium ions is larger than the interlayer spacing of graphite sheets, it is thus difficult for sodium ions to intercalate into graphite. In this regard, the excellent metal‐ion adsorption ability and rapid diffusion of sodium ions on the low‐dimensional GNR networks can be very promising in improving the capacities and power densities of SIBs, and deserve further research effort.

### 2.4 Developing NG‐Based van der Waals Heterostructures (vdWHs) for Efficient Optoelectronics

Besides the excellent optical and electronic properties of individual NG structures, NGs represent chemically tunable and fascinating molecular building blocks for constructing novel hybrid systems for versatile applications.^[^ ^131^, ^183^, ^184^ ^]^ In this context, the formation of supramolecular NG crystals or crystalline assemblies held together via non‐covalent forces such as *π*–*π* interactions has become an intensive research topic, with the aim to achieve high‐performance electronics and optoelectronics with favorable charge transport properties by tuning the intermolecular interactions. In this framework, development of the oxidative cyclodehydrogenation under electrochemical conditions led to direct large‐scale deposition of NGs with different morphologies,^[^ ^185^, ^186^ ^],^ e.g., elongated 1D conductive channels promising for notable electronics. Furthermore, the subtle design of the polyaromatic core and side‐chains offers exquisite control over the supramolecular self‐assembly process, yielding tunable electronic properties of the assemblies,^[^ ^187^ ^]^ including ad hoc charge transfer and energy transfer characteristics for efficient organic electronics and photonics.^[^ ^188^ ^]^ Furthermore, NGs decorated with long aliphatic chains in the peripheral positions form columnar liquid‐crystalline phases, which are stable from room temperature to over 400 °C. By taking advantage of these characteristics, temperature can be used as an effective knob to tune the degree of order at the supramolecular level in NG‐based films for enhanced optoelectronic performance.^[^ ^3^, ^189^ ^]^ We refer the readers to several recent reviews^[^ ^190^, ^191^, ^192^ ^]^ in which the supramolecular chemistry and resultant collective properties of NGs for electronics have been comprehensively summarized.

**Figure 6:**
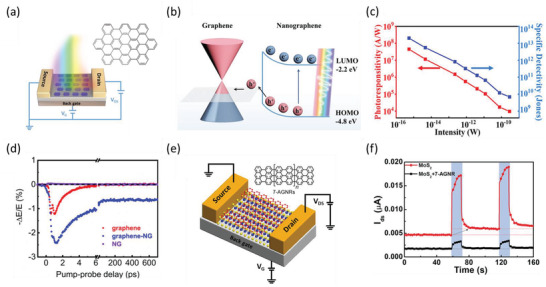
CT at NG‐2D materials vdW interfaces. a) Schematic of the photodetector with a FET configuration comprising graphene‐NG vdWHs in the device channel. The inset shows the chemical structure of the employed NG: C_60_H_22_. b) Energy diagram of the NG‐graphene vdWHs. After illumination, photogenerated holes transfer from NG to graphene, while the photogenerated electrons remain in the NG layer.^[^ ^196^ ^]^ c) Photoresponsivity and specific detectivity change at different optical illumination powers following 500 nm excitation. d) THz photoconductivity dynamics for graphene, NG‐graphene vdWHs, and NG following 400 nm excitation. e) Schematic of the FET device composed of MoS_2_‐GNR vdWHs. The inset shows the chemical structure of 7‐AGNR.^[^ ^189^ ^]^ f) Dynamic photoresponse of source–drain current upon 530 nm illumination (blue box area) for FETs based on pristine MoS_2_ (red curve) and MoS_2_‐GNR vdWHs (black curve) at *V*_g_ = 0 V.^[^ ^189^ ^]^ a–d) Reproduced with permission.^[^ ^196^ ^]^ Copyright 2021, American Chemical Society. e,f) Reproduced with permission.^[^ ^189^ ^]^ Copyright 2020, Wiley‐VCH.

Besides the formation of supramolecular NG crystalline structures, an emerging field related to the NG assembly lies in exploiting interfacial vdW interactions, e.g., between NGs and 2D materials, to construct hybrid organic‐inorganic vdWHs for exploring new photophysical functionalities. In particular, given the large library of existing NGs and their structural tunability by synthesis, one can envision programming the functionality of NG‐2D vdWHs by tuning the molecular structures and compositions. Furthermore, compared to conventional inorganic‐inorganic 2D‐2D vdWHs, the organic‐inorganic nature of NG‐2D vdWHs allows readily control of the interfacial coupling between the adjacent layers via solution‐processed procedures. This enables fine‐tuning of interfacial vdW interactions as ruled by the principles of molecular self‐assembly, and thus offers programmable electronic coupling through the selection of the NG dimension,^[^ ^193^ ^]^ composition,^[^ ^194^ ^]^ doping level,^[^ ^195^ ^]^ and deposition methods. Such a unique feature offers a powerful knob to modulate the interfacial electronic coupling and thus charge transport properties of the hybrids for optoelectronics. For instance, in a recent study, we developed a solution‐processed approach enabling deposition of NGs onto graphene to form strongly coupled NG‐graphene vdWHs by taking advantage of the strong interlayer *π*–*π* interactions. Such a vdW sensitization scheme combines the strong light absorption in NGs, efficient interfacial charge transfer (CT) between the layers, and excellent electrical transport properties of graphene, making it ideal for photodetection (**Figure** **6**a).^[^ ^196^ ^]^ The resulting photodetector shows an ultrahigh responsivity of 4.5 × 10^7^ A W^−1^ and a specific detectivity of 4.6 × 10^13^ Jones (Figure 6b), comparable with the state‐of‐the‐art graphene‐based photodetectors.^[^ ^3^ ^]^ Ultrafast THz spectroscopy further interrogated the microscopic CT process by tracking the photoconductivity in the graphene layer. As shown in Figure 6c,d, hole injection from NGs to graphene results in a photoconductivity modulation beyond 1 ns. The long‐lived charge separation, together with the efficient hole injection (≈10%) across NG‐Gr interfaces, rationalizes the superior properties observed in the photodetectors. Furthermore, in a recent report by Yan et al.,^[^ ^197^ ^]^ such NG‐Gr vdWH concept has also been implemented for water splitting. As bifunctional electrodes, the NG‐Gr heterostructure has been shown to achieve efficient oxygen and hydrogen evolution reactions simultaneously, demonstrating the great potential of NG‐Gr vdWH for energy applications.

Besides controlling the interfacial charge transfer process, the vdW sensitization has also been demonstrated as a powerful strategy to modulate the electrical transport properties of 2D materials, holding great promise for multilevel memory devices, water splitting, and photodetection.^[^ ^189^, ^196^, ^197^ ^]^ For instance, Liu et al.^[^ ^189^ ^]^ fabricated MoS_2_‐GNRs vdWHs (Figure 6e) comprising 7‐AGNRs and monolayer MoS_2_ to suppress the remaining photoconductivity after the termination of illumination, i.e., the so‐called “persistent photoconductivity (PPC) effect,” as displayed in Figure 6f. The origin of this effect can be traced to the CT process at MoS_2_‐GNR interfaces, where 7‐AGNRs withdraw the redundant electrons from the intrinsically n‐doped MoS_2_, as suggested by the weakened trion spectral weight and the red‐shifted A_1g_ phonon mode in MoS_2_‐GNR vdWHs. Thanks to the suppression of the PPC effect, coupling MoS_2_‐GNR vdWHs with photochromic molecules enables the photomodulation of source–drain current up to 52% and four distinguishable output current levels, demonstrating their great potential for multilevel memory devices.

## 3 Summary and Outlook

In this minireview, we have summarized the fundamental optical, electrical, and electrochemical properties of bottom‐up synthesized NGs, and their applications in optoelectronics and energy storage. We have highlighted the unique combination of blinking effect and outstanding chemical stability of GQDs for super‐resolution imaging. We have also discussed how the GNR's excellent charge transport properties and strong exciton effects make them a fascinating family of highly conductive organic conductors for efficient optoelectronics. Thanks to the edge‐specific electrochemical and chemical activity, NGs are useful for electrochemical energy storage (e.g., battery) and production (e.g., photocatalysis). Based on the collective properties of NGs, we surveyed the emerging NG‐based hybrid organic‐inorganic vdWHs obtained via supramolecular self‐assembly for efficient optoelectronics.

**Figure 7:**
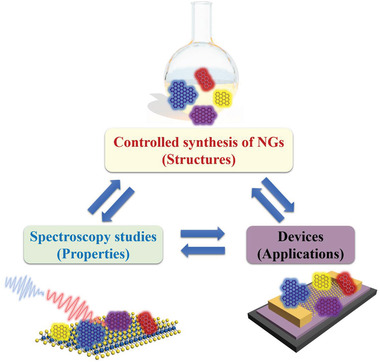
Demonstration of future efforts by combining controlled NG synthesis, spectroscopic studies, and device implementation, with an emphasis on revealing the structure–property–application correlation.

Future efforts in this field should aim at further improving the control over the physical and chemical properties of pristine NGs and at their hybrids when assembled into vdWHs (See **Figure** **7**). To achieve the first goal, substantial synthetic effort is required to finely tune the molecular structures of NGs, including the edge structures, length, side chains and heteroatoms, to preprogram the physical and chemical properties of NGs. The development of advanced and scalable deposition methods that can preserve the intrinsic properties of NGs once integrated into devices is critical for applications. Furthermore, although a few showcased studies have unveiled the potential of solution‐processed vdW engineering for efficient optoelectronics, such investigations only scratch the surface of the rich possibilities of NG‐based vdWHs for new functionalities. Further endeavors in exploring practical approaches to precisely control vdW interactions in NG assembly are essential to modulate the interfacial charge carrier dynamics for applications. In this aspect, tuning the size^[^ ^193^ ^]^ and composition^[^ ^194^ ^]^ (e.g., the relative ratio between the number of hydrogen and carbon atoms) of NG may be a fruitful approach to control the interfacial vdW interactions and thus coupling strength. In a recent theoretical study,^[^ ^193^ ^]^ it has been shown that vdW interactions in carbon‐based nanomaterials follow different scaling laws depending on the dimensionality and size, with vdW coefficients increasing as a function of size while decreasing as a function of layer number. Within this framework, interfacing NGs with materials with large lateral sizes and atomically thin thickness would facilitate the formation of strongly coupled NG assembly. Furthermore, modulation of charge carrier density in graphene has also been reported as an important variable to manipulate vdW interactions by changing the spatial extent of *π* orbitals.^[^ ^195^ ^]^ This makes the electrostatic control of the interfacial coupling possible and deserves further research effort. Furthermore, a deep understanding of the underlying mechanisms that determine the fundamental properties of NGs is required for advanced device integration and optimization. For instance, further spectroscopic investigations on the environment‐independent blinking properties in GQDs, the fascinating many‐body effects in GNRs, and the charge storage mechanism in NGs down to the atomic level are all critical aspects for NGs's applications in optoelectronic and energy applications. The latter will require advanced nanofabrication protocols for optimal interfacing and integration of all components to ultimately leverage high‐performance devices into new disruptive technologies.

## Conflict of Interest

The authors declare no conflict of interest.
